# Modified port placement and pedicle first approach for laparoscopic concomitant cholecystectomy and splenectomy in children

**DOI:** 10.4103/0971-9261.71750

**Published:** 2010

**Authors:** Kamalesh Pal

**Affiliations:** Department of Surgery, King Fahad Hospital of the University, College of Medicine, University of Dammam, Al Khobar, Kingdom of Saudi Arabia

**Keywords:** Cholecystectomy, splenectomy, laparoscopy

## Abstract

**Aim:**

Laparoscopy is becoming the preferred modality for concomitant cholecystectomy and splenectomy (CAS). Usually, six to seven ports are employed for CAS, and spleen is removed by classical lateral approach or anterior approach. We report here our modified five-port and pedicle first approach for CAS in children to minimize the intraoperative bleeding and maximize the access.

**Materials and Methods:**

Twenty-one children underwent laparoscopic CAS with this new approach and their data were recorded prospectively. Following cholecystectomy (with ports 1–4), left side was elevated by 30°. The spleen was lifted by a grasper/fan retractor through port no. 5. The pedicle was dissected and splenic vessels were divided by ligasure (vessels < 8 mm), and for bulkier pedicle, vascular endo-GIA stapler was used. Short gastric and gastrosplenic ligament, lower pole and phrenico-colic attachments and upper pole attachments were dissected by ligasure in that sequence. Spleen was placed in endosac and delivered by digital fracture technique. Occasionally, lower transverse incision was made to deliver a massive spleen.

**Results:**

There were 12 males and 9 females with an average age of 8 years. Fourteen had sickle cell disease (SCD) and 7 had SCD and beta thalassemia. All CAS were completed successfully without any complication. Total duration was 160 minutes. Cholecystectomy took an average of 35 minutes. Average blood loss was 140 ml. The mean splenic weight was 900 g and mean length was 20 cm. Duration of hospitalization was 3–4 days.

**Conclusion:**

CAS can be successfully performed by five ports. The pedicle first approach is extremely helpful in moderate to massive spleens as it reduces splenic size, vascularity and bleeding from capsular adhesions or inadvertant lacerations.

## INTRODUCTION

Laparoscopy is becoming the procedure of choice for cholecystectomy and splenectomy in children. Concomitant cholecystectomy and splenectomy (CAS) is often indicated in children with hematological conditions such as sickle cell disease (SCD), spherocytosis and thalassemia.[1–3] Port placement for standard cholecystectomy is different in children (<13 years of age) than adults and varies according to the age, size of abdomen and associated hepatomegaly[4–6] Although lateral approach is considered superior in mild to moderate spleens, bulky spleens (>700 g and >15 cm length) often create problems in dealing with hilar and upper polar dissections.[6,7] In SCD, increased parenchymal friability due to frequent sequestration crises[8] and perisplenic adhesions due to perisplenitis pose additional difficulty, often leading to capsular bleed, parenchymal lacerations, fractures and conversions to open surgery. The number of ports was reduced to five and positions of the ports were modified to aid in CAS. This prospective descriptive study analyses the feasibility and outcome of this novel approach to CAS in bulky spleens concomitantly with cholecystectomies in a position suitable to handle both the surgeries.

## MATERIALS AND METHODS

**Figure 1:**
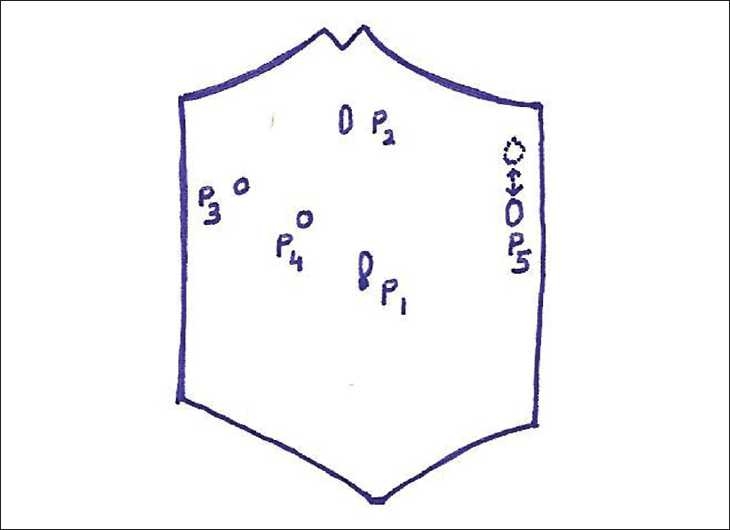
Schematic diagram of port placement. P1 = 10 mm umbilical port; P2 = 12 mm epigastric port; P3 = 5 mm right subcostal port; P4 = 5 mm midway P3–P1 port; P5 = 5–15 mm left subcostal port (15 mm for vascular endo-GIA stapler). Position of P5 varies according to the degree of splenic enlargement

Twenty-one children underwent laparoscopic CAS with this new approach and their data were recorded prospectively. The patient was in supine position, nasogastric (NG) tube was inserted to decompress the stomach and pneumoperitoneum was created by Veress needle. The port (P) placement is shown in Figure 1. Following cholecystectomy (with P1–4), left side was elevated by 30° and head end up by 15°–30°. The spleen was lifted by a grasper/fan retractor through port no. 5 (P5). A five-step pedicle first approach was employed as follows:

Step 1: Pedicle was dissected and splenic vessels were divided by ligasure (Valleylab, UK) for vessels <8 mm in diameter. For bulkier pedicle, vascular endostapler (Endopath, Ethicon, Cincinnati, OH, USA) was used through 15 mm port. Splenic artery was isolated and divided by ligasure.
Step 2: Short gastric and gastrosplenic attachments were scored by ligasure.
Step 3: Lower pole and phrenicocolic attachments were scored.
Step 4: Upper pole attachments were freed.
Step 5: Spleen was placed in endosac (EndoCatch II, US Surgical Corporation, Norwalk, CT, USA) and delivered by digital fracture technique. Occasionally, lower transverse incision was made to deliver a massive spleen. A drain was left in subphrenic space for 24 hours. Duration of surgery, amount of blood loss, any intraoperative or postoperative complications were noted prospectively.

## RESULTS

There were 12 males and 9 females with an average age of 8 years (6–13 years). Fourteen had SCD, and seven had SCD and beta thalassemia. All CAS were completed successfully without any complication. Total duration was 160 minutes (140–210 minutes). Cholecystectomy took an average of 35 minutes (25–45 minutes). In five cases, accessory spleen was found and removed successfully. Average blood loss was 140 ml (100–250 ml). Mean splenic weight was 900 g (750–1200 g) and mean length in the mid longitudinal axis was 20 cm (17–26 cm). Duration of hospitalization was 3–4 days. There were no postoperative complications such as subphrenic collection, organ injury prolonged ileus or vomiting. In all cases, NG tube and subphrenic drain were removed within 24–36 hours.

## DISCUSSION

Recently, laparoscopy has emerged as a safe and an effective modality for CAS.[2,3] Although laparoscopic splenectomy is the preferred modality for elective removal of normal sized to moderately enlarged spleens, massive spleens pose significant difficulty.[9,10] Although some authors have experienced clear advantage with right lateral position in dealing with mildly enlarged spleens, they have reported increased difficulty once the splenic size increases.[5,6,11] Bulky spleens are associated with higher blood loss, increased operating time and higher conversion rates.[12]

Parenchymal friability in situations like sequestration crisis, hemolytic crisis and perisplenic vascular adhesions leads to inadvertent parenchymal lacerations and troublesome hemorrhage.[8] In a lateral position, entire weight of the spleen drooping on a retractor (usually an endograsper) may lead to deep parenchymal laceration or even fracture of the pole and torrential hemorrhage. Some of the adjuncts described in adults to deal with such bulky spleens include preoperative splenic artery embolization or balloon occlusion,[13] *in situ* morcellation[14] and hand assisted laparoscopic surgery (HALS).[7,10,15,16]

Laparoscopic ligation of splenic artery in the lesser sac has been described to reduce the vascularity and aid in the removal of massive spleens;[17,18]

Early ligation of hilar vessels in the splenic pedicle significantly reduces the splenic size, vascularity and operative blood loss in open technique. To replicate this advantage of open technique, a pedicle first approach was employed in 21 consecutive cases of laparoscopic CAS in bulky spleens (mean weight = 900 g, mean length = 20 cm). Dissection of splenic hilum and division of vessels were technically easier, and mostly splenic artery could be ligated separately, except in five cases where bulky pedicle was divided *en bloc* by the application of endo-GIA vascular stapler. In cases with prominent splenic vein, additional clips were applied for security. The left oblique position prevented drooping of spleen until most of the vascular attachments were taken down. Higher placement of left subcostal port (P5) aided in dissecting the superiormost attachments. The modified placement of two right ports (P3 and P4) used for cholecystectomy helped in retracting left lobe of liver and stomach during the scoring of short gastric vessels and upper polar attachments. There were no conversions to open technique. Inadvertent parenchymal injuries or adhesions did not produce troublesome bleeding, and average blood loss was 140 ml, well below the others.[2]

Use of ligasure in all our cases had significantly eased the dissection with mean duration of combined procedure being 160 minutes (mean duration of cholecystectomy = 35 minutes). The upper pole of spleen was often found to produce a pouch (below the left diaphragm) and was difficult to access through standard port positions. Higher placement of port no. 5 close to left subcostal margin aided in the upper polar dissection in our cases. All the CAS were successfully completed with five ports without any intraopeartive or postoperative complications.

## CONCLUSIONS

Pedicle first approach is a safe and effective approach for CAS, including moderate to large spleens encountered in hemoglobinopathies. It gives the benefits of conventional splenectomy by early control of splenic vessels, thereby reducing the blood loss. Modified port placement reduces the number of total ports to five for CAS, helps in retraction of stomach and left lobe of liver, and improves the access to upper polar attachments in massive spleens. Left oblique position reduces the drooping of bulky spleen on the retracting instruments and attendant risk of parenchymal lacerations/fractures, especially in friable spleens, and yet keeps the bowel away from the field.

Further experience with this novel approach is needed to explore its advantages.
